# A scoping review of farm-level biosecurity measure effectiveness against foot-and-mouth disease to inform planning and preparedness efforts in the United States

**DOI:** 10.3389/fvets.2026.1819419

**Published:** 2026-06-08

**Authors:** MaRyka R. Smith, Christy J. Hanthorn, Michael W. Sanderson

**Affiliations:** Center for Outcomes Research and Epidemiology, College of Veterinary Medicine, Kansas State University, Manhattan, KS, United States

**Keywords:** biosecurity, cattle, foot-and-mouth disease, goat, scoping review, sheep, swine

## Abstract

**Introduction:**

Foot-and-Mouth Disease (FMD) is a global threat to cloven-hooved livestock and wildlife species. The causative agent, Foot-and-Mouth Disease Virus (FMDV) can be transmitted via aerosols, droplets, and fomites including humans. This makes the need for biosecurity measures that interrupt FMDV transmission during an outbreak critical. However, quantitative data supporting the effectiveness of many recommended biosecurity measures is limited. The purpose of this scoping review was to identify peer-reviewed literature that described or evaluated biosecurity practices to mitigate FMD risk at the farm level in order to inform planning and preparedness efforts in the United States.

**Methods:**

A systematic search of four databases identified 3,153 unique records. Included reports had to be original, peer-reviewed research, published in English, include information about a biosecurity measure applied at the farm level to mitigate FMD risk, and address at least one major domestic livestock species in the United States (cattle, sheep, goat, swine, American bison). Reports describing results generated entirely *in-silico* or with surrogate viruses were not included.

**Results:**

22 reports were included in this review describing data from 16 (one unreported) unique countries published between 1998 and 2024. Two reports describing experimental studies evaluated handwashing, showering, and changing outerwear, which were found to have variable efficacy. The use of biosecurity measures applied to livestock and their products, personnel and visitors, vehicles and fomites, and disinfection were identified from 20 field reports and observational studies. No biosecurity measures that appeared in more than one report were identified as being consistently effective at mitigating disease risk in a field setting.

**Discussion:**

The inconsistent and limited evidence to support the effectiveness of specific biosecurity measures in field settings identified through this review makes meta-analysis infeasible at this time and hampers the ability of animal health officials and animal caretakers to plan for and respond appropriately during an FMD outbreak. Improved reporting of biosecurity measure implementation would improve our ability to assess their effectiveness and develop evidence-based biosecurity recommendations for FMD preparedness and response plans.

## 1 Introduction

Foot-and-mouth disease (FMD) is a viral vesicular disease that impacts cloven-hooved livestock in many areas of the world (1). The global annual costs of FMD have been estimated at $6.5 to 21 billion in endemic regions and an additional $1.5 billion or more in free countries and zones (2). FMD is highly contagious and easily transmissible between farms and among animals. The virus is shed in all bodily excretions and secretions with many animals becoming infectious prior to the onset of clinical signs (3). The virus can also be spread via aerosolized particles and on fomites such as humans and vehicles (4). The ease of transmission, highly contagious nature, and global cost of FMD make effective biosecurity measures a critical, albeit difficult to implement, component in controlling an outbreak.

Biosecurity measures are, “the implementation of a segregation, hygiene, or management procedure (excluding medically effective feed additives and preventive/curative treatment of animals) that specifically aims at reducing the probability of the introduction, establishment, survival, or spread of any potential pathogen to, within, or from a farm, operation or geographical area” (5). Globally, producers, animal health officials, and veterinarians utilize many different biosecurity measures collectively to control outbreaks of FMD; however, quantitative data on the effectiveness, or ineffectiveness, of individual biosecurity measures in the scientific literature is lacking. Limited opportunities exist to evaluate biosecurity measure effectiveness during FMD outbreaks, and conducting research on FMD in countries that are free from the disease is challenging. In many free countries, including the United States (U.S.), FMD research is limited to high-containment, national laboratory facilities. Thus, researchers must rely on original research from FMD endemic or outbreak countries and reviews of global data to gather field-tested information that can be used to inform FMD response plans in the U.S.

The U.S. has been free from FMD since 1929, but the volume of global trade and livestock movement heighten the concern of a potential foreign animal disease re-introduction, as has been seen with recent FMD introductions to countries that had previously eradicated the disease (1, 6). In the event of an FMD incursion, the current United States Department of Agriculture (USDA) plans include the likely use of controlled movement orders and standstills that would temporarily halt movement of susceptible livestock and fomites (7). These orders will most likely originate from federal animal health officials and be implemented at various levels of jurisdiction (state, local) depending on the location, type, and phase of the outbreak (8). The cessation of animal movement, even temporarily, could prevent further FMD spread but would also negatively impact the continuity of business for producers whose livestock are not infected with FMD. To address this negative impact, methods to allow permitted or low-risk movement of animals are under development (9). The Secure Beef Supply (SBS) plan has been developed to aid in the continuity of business in the face of an outbreak through the use of enhanced biosecurity and animal monitoring. The current SBS plan recommends identifying a Biosecurity Manager, actively monitoring animals for clinical signs of disease, creating a line of separation with access points to the animal areas, and implementing enhanced biosecurity measures, such as cleaning and disinfection stations (10). In other developed countries, including (but not limited to) Australia and Canada, the plans for response to and control of FMD outbreaks include similar elements of biosecurity measure implementation (11, 12). The recommended biosecurity measures in these plans are best practices for decreasing the likelihood of FMD introduction and spread among farms, but data on the effectiveness of any specific procedure is sparse in peer-reviewed literature sources. To address this knowledge gap, a scoping review was chosen as the preferred method to gather and summarize the breadth of information on biosecurity measure effectiveness in endemic or outbreak settings. To the authors' knowledge, this review is the first of its kind to comprehensively summarize data on individual biosecurity measure effectiveness in decreasing FMD risk.

The aim of this scoping review was to identify, from peer-reviewed literature, biosecurity measures that have been described and/or evaluated for effectiveness in preventing or controlling FMD introduction or spread at the farm or animal level in order to inform U.S. planning and preparedness efforts. Identifying and addressing the data scarcity regarding estimates of biosecurity measure effectiveness at the farm-level can help researchers plan targeted research and develop more accurate disease spread models, U.S. animal health officials improve plans and policies, and veterinarians and producers to make informed decisions to prevent and control FMD. Such information could also be utilized beyond the U.S. to aid FMD response plans globally. Further, the ability to estimate the potential economic value of the recommended biosecurity measures would be informed by data on their effectiveness.

## 2 Methods

This scoping review was conducted according to the Preferred Reporting Items for Systematic Reviews and Meta-Analyses extension for Scoping Reviews (PRISMA-ScR) (13). The objective was developed by group discussion as part of a larger review project; therefore, the expanded scope is reflected in the search strategy. A formal protocol for this scoping review was not developed *a priori*.

### 2.1 Information sources and search strategy

The search strategy was developed by the reviewers in collaboration with a librarian within Kansas State University's College of Veterinary Medicine. Inclusion of the phrase “foot and mouth”, but not “hand foot and mouth,” in the title or abstract was required as well as at least one of the variations of topic keywords relevant to biosecurity or the wider search being conducted by the reviewers (e.g., surveillance, transmission). The search was completed in two steps with an initial search in 2021 and an update in 2024. Detailed search strategies and results are presented in Table 1. Both the initial and updated searches were limited to records available in English but were not limited by date.

**Table 1:** Search strategies by database and number of records returned by date.

Database and date range of coverage	Search string	Number of records from Sept 29, 2021	Number of records from June 5, 2024
CAB 1973 - present	(ab:(“foot and mouth”)OR title:(“foot and mouth”)) AND ab:(surveill^*^ OR transmi^*^ OR persist^*^ OR biosecur^*^ OR disinfect^*^ OR “virus surviv^*^” OR “environment^*^ surviv^*^” OR bird OR pest OR rodent OR wildlife) NOT ab:(“hand foot and mouth”) Language=English	1,778	Not included
Scopus 1800s - present	(TITLE-ABS (“foot and mouth”)) AND ABS (surveill^*^ OR transmi^*^ OR persist^*^ OR biosecur^*^ OR disinfect^*^ OR “virus surviv^*^” OR “environment^*^ surviv^*^” OR bird$ OR pest$ OR rodent$ OR wildlife) AND NOT ABS (“hand foot and mouth”) AND (LIMIT-TO (LANGUAGE, “English”))	1,446	1,690
Web of Science 1800s - present	((TI=(“foot and mouth”) OR AB=(“foot and mouth”)) AND AB=(surveill^*^ OR transmi^*^ OR persist^*^ OR biosecur^*^ OR disinfect^*^ OR “virus surviv^*^” OR “environment^*^ surviv^*^” OR bird$ OR pest$ OR rodent$ OR wildlife) NOT AB=(“hand foot and mouth”)) AND LA=(English)	1,238	2,066
PubMed 1950 – present	(“foot and mouth” [Title/Abstract] AND(surveill^*^ [Title/Abstract] OR transmi^*^ [Title/Abstract] OR persist^*^ [Title/Abstract] OR biosecur^*^ [Title/Abstract] OR disinfect^*^ [Title/Abstract] OR “virus surviv^*^” [Title/Abstract] OR “environment^*^ surviv^*^” [Title/Abstract] OR bird^*^ [Title/Abstract] OR pest^*^ [Title/Abstract] OR rodent^*^ [Title/Abstract] OR wildlife [Title/Abstract]) NOT (“hand foot and mouth” [Title/Abstract]) AND english [la])	1,301	1,508

The search strings utilized were optimized for each search engine's unique Boolean logic requirements.

For the initial search performed on September 29, 2021, authors utilized CAB Abstracts (via CAB Direct), Scopus, Web of Science, and PubMed databases. These databases were selected based on the findings in Grindlay, et al. and their availability to the reviewers via the Kansas State University library system (14). For the search update conducted on June 5, 2024, authors utilized PubMed, Scopus, and Web of Science. The same search strings were used for each database for both the original and updated search; however, the search algorithm in CAB was no longer compatible with the original search string; therefore, an updated search in CAB was not included. The records identified with the 2024 search update were deduplicated against all records returned by the 2021 search. Hand searching of literature was not utilized for this review.

### 2.2 Screening process

The records returned by the databases were exported as RIS files and imported to R statistical software (15). The package RevTools was then used to identify and remove duplicate records based on the DOI number and title (16). The remaining records were imported to Zotero which identified and consolidated further duplicate records (17). Resultant records were exported to Microsoft Excel for screening (18). Titles and abstracts were screened in duplicate with one reviewer (MRS) screening all titles and abstracts and two other reviewers (CH, MWS) each screening half of the total titles and abstracts. Any disagreements on a specific record were discussed between the two assigned reviewers and if an agreement was not reached, the third reviewer was consulted to achieve consensus. Following title and abstract screening, full-texts of reports were sought. All retrieved reports were screened following the same process of reviewing in duplicate. Both screening processes utilized a screening tool developed by the reviewers. The tool was piloted on a subset of records and reports for quality and agreement with adjustments being made as needed. Questions included in the screening tool were:

(1) Is the record or report available in English?
(2) Does the record or report describe Foot and Mouth Disease?
(3) Is the report original work published in a peer-reviewed journal?
(4) Does the report describe whole animal studies (*in vitro* studies excluded)?
(5) Does the report describe outcomes for topics other than vaccination strategies or development, virus diagnostics, viral genetics, or immune response?
(6) Is the data reported from non-simulated studies? (computer simulations or estimations excluded)
(7) Does the report describe or evaluate a biosecurity measure applied at the farm level?
(8) Does the report include outcomes for at least one of the U.S. domestic livestock species of interest (cattle, goats, sheep, swine, American bison)?

Screening questions could receive a “yes” or “no” answer. If the record or report received two “no” responses to any single question from both reviewers it was immediately excluded. If the record or report received a “yes” to all questions from both reviewers it was included in the next review step. The search update utilized the same tools as the original search and screening process.

### 2.3 Eligibility criteria

To be included in this review, reports had to fulfill the screening criteria described above. Reports that only addressed wildlife or livestock species not common to the U.S. (e.g., African buffalo) were excluded in order to align with the aim of the review to inform U.S. planning and preparedness efforts. *In-vitro* studies, immunology studies, pathology studies (including challenge models), studies which utilized surrogate viruses, surveillance reports, and reports that utilized vaccination as the sole means of FMD control were excluded. Reports that only contained data from mathematical or simulation models were excluded. Authors needed to evaluate or describe biosecurity measures intended to be implemented at the farm level rather than measures implemented nationally, regionally, or off-site for a report to be included. Generalized biosecurity recommendations for livestock industries (such as for exhibition) were excluded. Biosecurity measures not included in this review were the utilization of vaccination or culling as the sole means of disease control and national or regional animal movement controls. No date or geographic location restrictions were applied to reports.

### 2.4 Data charting and synthesis

For all reports included in the review, data were charted by a single reviewer (MRS) and verified by a second reviewer (CH). Key data items were charted at the report level and are summarized in Table 2. Citation information, country and year(s) of data collection, animal species and use, and biosecurity measure descriptions were charted as reported by the authors. If available, biosecurity measure effectiveness was charted as reported by report authors, or if sufficient information was available, was summarized by reviewers. Biosecurity measures which were both evaluated and demonstrated to be efficacious or effective are presented separately from biosecurity measures that did not have data to support their effectiveness. Biosecurity measures that were only described as being implemented and those that lacked data to demonstrate effectiveness were categorized by reviewers into: (1). biosecurity measures for livestock and livestock product movement, (2). biosecurity measures for personnel and visitors, (3). biosecurity measures for fomites, and (4). disinfection measures. Biosecurity measures for livestock and their products included management policies for susceptible livestock species of interest (e.g., isolation and quarantine) and their products (e.g., carcass, manure, and milk disposal). Biosecurity measures for personnel and visitors included personal protective equipment use and management policies regarding workers and/or visitors (e.g., access to animal areas). Biosecurity measures for fomites included management policies for vehicles (e.g., designated parking areas), non-livestock species management, and management strategies for other routes of indirect viral transmission. Disinfection measures included disinfection strategies for clothing, vehicles, and facilities to limit viral spread as reported by authors. Data are presented in tabular or text format.

**Table 2:** Key data items.

Data item	Description
Citation information	List of authors, article title, journal title, publication date
Location	Country of data collection as reported by the authors
Study date	Year(s) of data collection as reported by the authors
Animal species and use	Study population species (cattle, goats, sheep, swine, American bison) affected by implemented biosecurity measures. Use was charted for cattle as beef (includes “fattening”), dairy, both, or unspecified.
Biosecurity measure description	Descriptions of individual biosecurity measures were captured as described by the authors and subsequently categorized as follows: 1) Biosecurity measures for livestock and their products included management policies for susceptible livestock species of interest (e.g., isolation and quarantine) and their products (e.g., carcass, manure, and milk disposal) 2) Biosecurity measures for personnel and visitors included personal protective equipment use and management policies regarding workers and/or visitors (e.g., access to animal areas) 3) Biosecurity measures for fomites included management policies for vehicles (e.g., designated parking areas), non-livestock species management, and management strategies for other routes of indirect viral transmission 4) Disinfection measures included disinfection strategies for clothing, vehicles, and facilities to limit viral spread as reported by authors
Biosecurity measure effectiveness	Description of biosecurity measure effectiveness if reported by authors or as summarized by reviewers if sufficient information was available

The key data items that were charted for all included reports included citation information, country, and year(s) of data collection. Animal species and use was charted as reported by the authors. Biosecurity measure descriptions were charted as reported by the authors, and subsequently categorized by reviewers. If available, biosecurity measure effectiveness was charted as reported by report authors, or if sufficient information was available, was summarized by reviewers.

## 3 Results

### 3.1 Selection of sources of evidence

**Figure 1:**
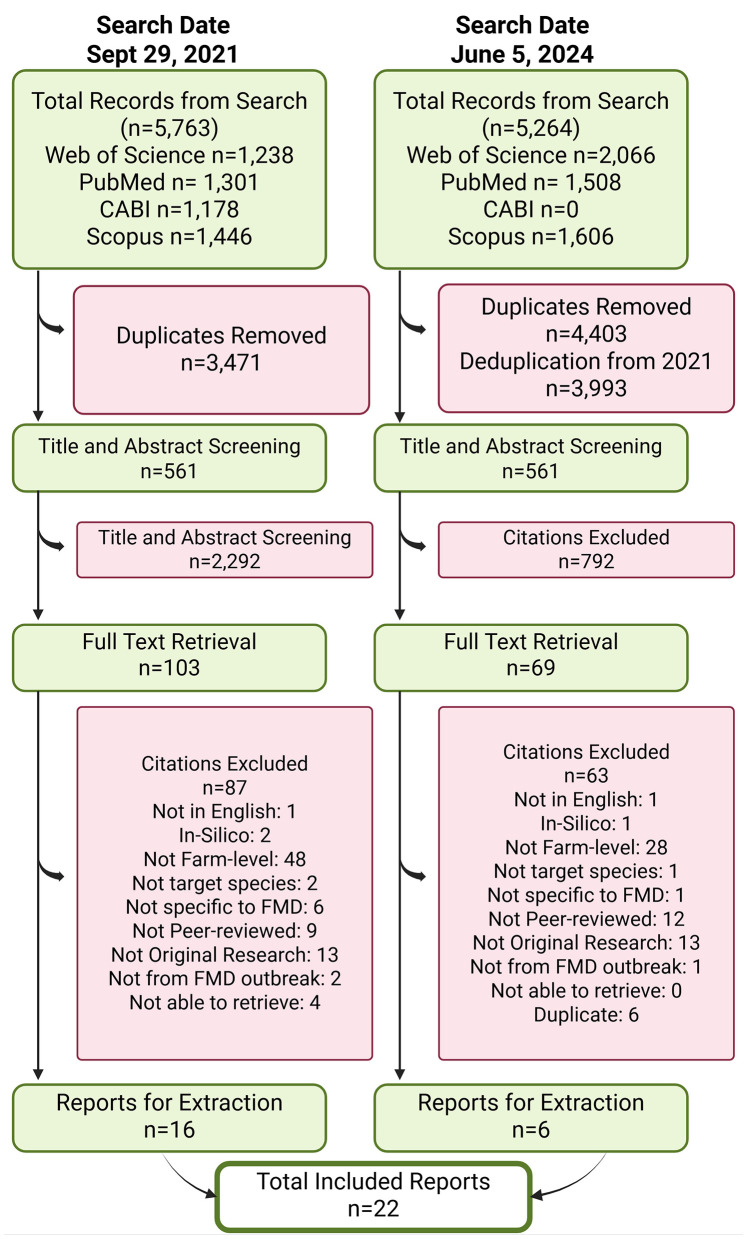
Flow diagram of the number of records and reports identified, screened for eligibility, and retained for inclusion in the review from an original and an update search. Eligibility screening utilized a title and abstract screen followed by a full-text screen. Reasons for report exclusion during full-text screening are detailed.

Results of the two searches and screening processes are summarized in Figure 1. The 2021 search of the four databases returned 5,763 records. After 3,475 duplicate records were removed, 2,291 records were eligible for title and abstract screening. Following title and abstract screening, 103 reports were sought for full-text retrieval and further screening. Four reports could not be retrieved. Of the 99 reports evaluated for eligibility, 83 were excluded based on the criteria detailed in Figure 1. 16 reports met the eligibility criteria for inclusion in this review.

The 2024 search of three databases returned 5,264 records. Following deduplication, 861 records were eligible for title and abstract screening where 792 were excluded. The remaining 69 reports were sought for retrieval and further screening. All 69 reports were retrieved, and 6 met the eligibility criteria for inclusion in this review. A total of 22 reports identified from the two searches were charted.

### 3.2 Characteristics of reports

Report characteristics are summarized in Table 3, including susceptible species (and use), country and year of data collection, and reference. The 22 included reports described FMD data collection between 1952 and 2022 from 16 countries, with one report not specifying the country of data collection. No reports were identified that addressed FMD biosecurity measures for American bison, but all other species of interest were represented. Reports that addressed FMD biosecurity measures for cattle were most common (*n* = 18), followed by swine (*n* = 8), sheep (*n* = 6), and goats (*n* = 3). Of the 18 reports with cattle in the study population, dairy cattle alone were included in 6 reports, beef cattle alone were included in 2 reports, beef and dairy cattle were included in 2 reports, and 8 reports did not specify the type of cattle. Reports included in the review were published in 12 different journals. The journals most frequently represented were *Preventative Veterinary Medicine* and *Frontiers in Veterinary Science* each with 4 included reports, and *Veterinary Record* and *Transboundary and Emerging Diseases* each with 3 included reports. No reports were identified where biosecurity measures were described with data demonstrating effectiveness, but two included reports described biosecurity measures with accompanying data to demonstrate efficacy. The remaining 20 included reports either simply described biosecurity measures implemented or had data that did not support the biosecurity measure's effectiveness.

**Table 3:** Report characteristics.

Species (use)	Country; Year(s) of data	Reference
Cattle (beef)	Australia, 2017–2018	Manyweathers, et al. (31)
	England, 2007	Ellis-Iverson, et al. (37)
Cattle (dairy)	Egypt, 2012	Byomi (21)
	Ethiopia, 2021–2022	Seifu et al. (33)
	Saudi Arabia, 1994	Hutber and Kitching, (30)
	Thailand, 2016–2017	Sansamur, et al. (39)
Cattle (unspecified)	Cambodia, 2015	Young, et al. (28)
	Ethiopia, 2021	Shurbe, et al. (26)
	Pakistan, 2019	Ali, et al. (23)
Cattle (beef and dairy) and swine	Korea, 2014–2019	Lee, et al. (40)
	USSR Estonia, 1952, 1982	Kass, et al. (35)
Cattle (dairy), goats, and sheep	Kenya, 2012	Lyons, et al. (22)
Cattle (dairy) and swine	Korea, 2002	Wee, et al. (36)
Cattle (unspecified), goats, and sheep	Mongolia, 2021	DiPietro, et al. (27)
Cattle (unspecified), goats, and swine	Lao People's Democratic Republic, 2016	Miller, et al. (25)
Cattle (unspecified) and sheep	Cameroon, 2016	Mielke, et al. (38)
Cattle (unspecified) and swine	Japan, 2010	Hayama, et al. (34)
	Lao People's Democratic Republic, 2010	Nampanya, et al. (24)
Sheep	Australia, 2017–2018	Manyweathers, et al. (32)
Sheep and swine	United States (PIADC^*^), NR	Amass, et al. (20)
	United States (PIADC^*^), NR	Amass, et al. (19)
Swine	NR^**^, 2002	Poulin and Christianson, (29)

Summary of report characteristics including species and use of study population, country and timeframe (year(s)) of data collection, and reference. NR, not reported.

^*^Plum island animal disease center.

^**^No specific country reported by authors.

### 3.3 Biosecurity measures demonstrated to be efficacious

Two included reports experimentally evaluated the efficacy of biosecurity measures for preventing FMD transmission (19, 20). Both reports describe studies conducted at the Plum Island Animal Disease Center (PIADC) in the United States. Readers are directed to the original reports for full study design and sample collection information. Both studies utilized FMD inoculated pigs as the source of infection and assessed transmission to sheep and/or pigs via human contacts. The researchers handled the pigs 2 days after inoculation, collecting various samples, then they moved to a room with five naïve animals of each species after implementing one of three levels of biosecurity measures. The first experimental group received no biosecurity intervention. Researchers directly contacted naïve animals after handling the infected animals, deemed “direct human exposure” by the authors. For the second group, researchers washed their hands and changed outerwear between animal rooms. For the third group, researchers completely changed their clothing and showered between animal rooms.

In their 2003 study, Amass et al. used the strain FMDV (O/UK/35/2001) which is known to infect both sheep and pigs (19). In the direct human exposure group, all animals (five pigs, five sheep) developed gross lesions consistent with FMD infection, and three of the five sheep seroconverted. In the hand washing and new outerwear group, three out of five sheep developed gross lesions, and three out of five sheep seroconverted, but none of the pigs (0/5) showed signs of clinical disease or seroconverted by the end of the study. In the shower and new outerwear group, none of the animals (0/5 pigs, 0/5 sheep) developed gross lesions or seroconverted.

In their 2004 study, Amass et al. used the FMDV (O/TAW/97) strain, which is known to infect pigs but not sheep, therefore only pigs were used to evaluate the biosecurity measures implemented in this study (20). In the direct human exposure group, all five of the pigs developed gross lesions consistent with FMD infection, and one of the pigs developed IgM antibodies. None of the pigs in either the handwashing and clean outerwear group (0/5) or the shower and clean outerwear group (0/5) developed gross lesions or seroconverted.

### 3.4 Other implemented biosecurity measures

Twenty reports described biosecurity measures that were implemented on farms specifically for FMD prevention or control, but the authors either did not evaluate the biosecurity measure for effectiveness or provided data that indicated the biosecurity measure was not effective. Biosecurity measures for livestock and their products were most common in the literature appearing in 15 reports. Biosecurity measures for personnel and visitors were identified in seven reports. Biosecurity measures for vehicles and other fomites were identified in six reports. Disinfection measures were identified in 11 reports.

#### 3.4.1 Biosecurity measures for livestock and their products

Reported biosecurity measures applied to livestock included isolating infected animals from the rest of the herd, physical separation and separate management of animal groups within a farm, isolating, quarantining, or inspecting livestock prior to herd introduction, and maintaining separation between herds. Biosecurity measures applied to livestock products included management strategies for transport and disposal of animal wastes (feces and carcasses) and products (milk). The number of reports where unique biosecurity measures for livestock and their products were implemented by their reported effectiveness is summarized in Table 4. Expanded descriptions for livestock and livestock product biosecurity measures and their effectiveness are reported in Supplementary Table 1.

**Table 4:** Number of reports where unique biosecurity measures for livestock and livestock products were implemented by their reported effectiveness.

Biosecurity measure	Number of unique reports				
	Beneficial	Detrimental	Neutral	Effect not determined	Total (*n* = 15)
Isolation of infected animals from the rest of the herd/group	0	2	1	5	8
Separation of animal groups within farm	3	0	2	0	3
Isolation, quarantine, or inspection of incoming stock	0	0	1	3	4
Managing farm as a closed herd	0	0	0	2	2
Covering carcasses for transport	1	0	0	0	1
Carcass disposal by burning	0	0	0	1	1
Feces stored instead of spread as fertilizer	0	0	0	1	1
Milk disposed of or pasteurized before feeding to other animals	0	0	0	1	1

A single report may discuss variable outcomes for individual biosecurity measures. Therefore, reports may belong to more than one effect category per row.

Isolating clinically ill animals was the most commonly described biosecurity measure for livestock, appearing in eight reports (21–28). Two included reports described implementation of isolation practices in such a way that it exacerbated disease spread. Byomi reported that the placement of isolation pens near a highway and without sufficient separation from other groups of livestock on the farm was a contributing factor for disease spread among cattle on a dairy in Egypt. Lyons et al. described a situation where the movement of infected cattle on a dairy in Kenya from their home paddock to an isolation paddock resulted in viral exposure of other groups of cattle on the farm, both from the movement of the infected cattle and from placing another group of cattle on the contaminated pasture. Ali et al. used a case-control study design to compare farms that did and did not isolate clinically ill animals. Isolation was significantly associated with being a case farm (*p* < 0.2) in a univariable model but was not retained in the final multivariable model. Isolating infected animals was only described and was not evaluated in the remaining five reports. Specific details on isolation practices were lacking in most of the reports included in this review. Miller et al. (25) reported that 31.7% (*n* = 20) of the households that they surveyed in the Greater Mekong Subregion of Lao People's Democratic Republic responded that they isolate clinically affected livestock until clinical recovery. No other reports specified a duration for isolation. No other reports, aside from those above, specified locations for the isolated livestock (25).

Utilizing spatial separation as a management strategy for groups of livestock within herds was described in three reports (22, 29, 30). Lyons et al. reported that small ruminants were kept on a separate pasture “a few kilometers” from the dairy cattle present on the farm, and Poulin and Christianson reported that apparently healthy piglets were transferred to a site 300 meters from their farm of origin following weaning. Both of these strategies appeared to be sufficient for preventing FMD transmission during the reported outbreaks. Hutber and Kitching described using pens of immune calves to separate clinical and healthy cattle on a dairy in Saudi Arabia, which also appeared to be sufficient for preventing FMD transmission during that outbreak. Conversely, Hutber and Kitching reported that the use of physical and spatial boundaries slowed disease transmission between pens of cattle but were not sufficient for preventing transmission. Additionally, Poulin and Christianson reported that moving apparently healthy sows from an infected farm to a separate site was not sufficient to prevent infection.

Four reports described utilizing quarantine, isolation, and/or inspection procedures for incoming livestock (23, 28, 31, 32). In their case-control study Ali et al. did not find a significant association between farms that utilized a quarantine measure for new herd introductions and disease risk. Details of the quarantine measures, including quarantine length and location were not reported. Young et al. reported that surveyed smallholder cattle farmers in Cambodia that experience epidemic waves of FMD responded that they always (26.3%), sometimes (25.8%), or never (47.9%) isolated incoming stock for 2 weeks; however, no estimate of preventive effectiveness was reported. Manyweathers, et al. surveyed Australian beef (2020) and sheep producers (2021) and found that the majority of survey respondents inspect or isolate incoming livestock, but these procedures were not evaluated for their effectiveness regarding disease prevention. Additionally, specific inspection and isolation protocols were not reported.

Two reports described efforts to maintain a closed herd, limiting contact with outside animals (28, 33). Seifu et al. reported that only a small percentage of surveyed dairy farmers in Ethiopia who were “aware of FMD” keep cattle within the farm compound (9.6%), prevent other cattle from entering their farms (6.3%), or do not bring new cattle to the farm (3.1%) to prevent FMD infection. Young et al. reported a that higher percentage of surveyed smallholder cattle farmers in Cambodia reported preventing their cattle from having direct contact with other farmers' cattle (19.2%) or keeping their cattle in a fenced area (78.3%). However, neither author evaluated the effectiveness of these biosecurity measures.

In a multivariable model for their retrospective case-control study, Hayama et al. reported that the following measures were not significantly associated with increased FMD transmission risk to Japanese cattle and pig farms: utilizing burial sites within 500 meters of farms or the presence of transportation roads within 200 meters. These risk factors were evaluated in conjunction with using waterproof sheets to cover carcasses during transport, transporting carcasses in sealed containers to burial sites, and disinfecting vehicles and equipment to prevent disease transmission (34). Kass et al. reported burning as a means of carcass disposal during an FMD outbreak in Soviet Estonia between 1982 and 1983. They also described the storage of manure and disposal of milk from infected animals during the outbreak but did not evaluate the effectiveness of these waste disposal methods on limiting disease spread (35).

#### 3.4.2 Biosecurity measures for personnel and visitors

Reported biosecurity measures applied to farm personnel and visitors included wearing personal protective equipment (PPE) such as disposable work suits, gloves, boot covers, or farm specific clothing and/or footwear. Additional biosecurity measures that impacted the management of personnel and visitors included restriction of visitor access, farm lockdowns, bathing, and specific assignment of animal caretakers to individual livestock groups. The number of reports where unique biosecurity measures for farm personnel and visitors were implemented by their reported effectiveness is summarized in Table 5. Expanded descriptions for farm personnel and visitor biosecurity measures and their effectiveness are reported in Supplementary Table 2.

**Table 5:** Number of reports where unique biosecurity measures for personal protective equipment and individual management were implemented by their reported effectiveness.

Biosecurity measure	Number of unique reports				
	Beneficial	Detrimental	Neutral	Effect not determined	Total (*n* = 7)
Farm-specific clothing reusable or disposable	0	0	2	1	3
Farm-specific footwear reusable or disposable	0	0	1	1	2
Gloves	0	0	0	1	1
Restriction of visitor access to farms	0	0	0	3	3
Farm lockdown	0	0	0	1	1
Bathing	0	0	0	1	1
Minimizing cross contact between animal groups	0	0	0	1	1

A single report may discuss variable outcomes for individual biosecurity measures. Therefore, reports may belong to more than one effect category per row.

Six instances of wearing PPE were described in three reports (35–37). Wee et al. described a situation from the 2002 FMD outbreak in Korea during which a worker who was part of swine culling activities was issued a disposable work suit and boot covers for the duration of the cull, and which were destroyed afterward. However, in the subsequent outbreak investigation, it was determined that the jeans worn underneath the work suit were still likely contaminated during the culling event and served as a source of contamination for the worker's car and eventually another site during the outbreak. Kass et al. also reported the use of protective clothing by dairy farm workers during the 1982 FMD outbreak in Soviet Estonia. However, since the district where this biosecurity measure was implemented did not experience any FMD cases, it is not possible to determine the effectiveness of the practice. No detail was given regarding the material or specifics of the clothing that was used. Additionally, Kass et al. reported that workers on dairies that did have FMD cases during Soviet Estonia's 1982 FMD outbreak used rubber gloves when handling sick animals and when milking animals with sores on their udders. No indication of the effectiveness of this practice was reported. Ellis-Iverson et al. used a case-control study to investigate the association between providing clothing or footwear to farm staff and being a case farm during the 2007 FMD outbreak in Southern England. Neither practice was significantly associated with the farms' FMD infection status. Details regarding the specific clothing and footwear provided to workers on each farm were not given, but it was noted that the clothing provided could include overalls and coats.

The management of farm personnel or visitors was mentioned in six reports, but none of the reported biosecurity measures were evaluated for effectiveness (22, 31–33, 35, 36). Restricting visitor access to farms was mentioned in three reports, all surveys of livestock producers, with only one survey being conducted in an FMD endemic location (31–33). Seifu et al. (33) reported that restricting visitor access to the surveyed dairy farms in Central Ethiopia, an area where FMD is endemic, is not widely practiced as only 2.1% of the surveyed dairy farmers reported doing so. Manyweathers, et al. surveyed Australian beef producers to assess vulnerability in the event of an FMD outbreak and reported that only 25% of the surveyed farms always restricted visitor access and 23% never restricted access. They also reported that over 39% of the surveyed beef farms did not require visitors to undergo any biosecurity practices. Biosecurity practices that were imposed by the remaining farms were not reported. In a similar study, Manyweathers, et al. surveyed Australian sheep producers and found that 32% of producers reported regularly restricting visitor farm access but over half (53%) of the surveyed farms responded that they did not require visitors to follow biosecurity practices.

The use of farm lockdowns, bathing, and specific assignment of animal caretakers to individual livestock groups were mentioned in one report each. Kass et al. (35) reported that strictly enforced, complete farm lockdowns for FMD positive herds were utilized during the 1952 and 1982 FMD outbreaks in Soviet Estonia. Wee et al. (2008), reported that public baths were used as part of a decontamination process for workers following the completion of culling events during the 2002 FMD outbreak in Korea (36). Lyons, et al. reported the assignment of stockmen to single animal groups on a large dairy in Kenya during an outbreak in an effort to minimize cross-contact among animal groups and reduce within-herd FMD spread (22).

#### 3.4.3 Biosecurity for vehicles and fomites

Biosecurity measures for fomites were identified in six reports and included control of non-livestock species, vehicle access control, and limiting shared equipment. The number of reports where unique biosecurity measures for fomites were implemented by their reported effectiveness is summarized in Table 6. Expanded descriptions for fomite biosecurity measures and their effectiveness are reported in Supplementary Table 3.

**Table 6:** Number of reports where unique biosecurity measures for fomite management were implemented by their reported effectiveness.

Biosecurity measure	Number of unique reports				
	Beneficial	Detrimental	Neutral	Effect not determined	Total (*n* = 6)
Feral animal management	1	0	0	2	3
Allowing dogs on farm	1	0	0	0	1
Designated vehicle parking	1	0	0	0	1
Barrier to vehicle entry	1	0	0	0	1
Not sharing equipment between farms	0	0	1	1	2

A single report may discuss variable outcomes for individual biosecurity measures. Therefore, reports may belong to more than one effect category per row.

Three reports addressed the control of feral animals (29, 31, 32). Poulin and Christianson reported that rodent control efforts were strengthened to prevent recontamination of cleaned areas during a successful FMD eradication process on a swine farm in Asia. Manyweathers, et al. found that approximately 20% of surveyed Australian beef producers never, rarely, or occasionally control feral animals on their farms, compared to their subsequent 2021 survey of sheep producers, where the percentage of producers who did have control plans for feral animals was slightly less than 30%.

In their case-control study of the 2007 U.K. FMD outbreak, Ellis-Iverson et al. investigated risk factors associated with FMD infection on secondary case farms (i.e., farms that became infected due to disease spread from primary case farms which were infected from the direct viral source). They reported, based on univariable *p*-values, that control farms were more likely than case farms to have biosecurity measures in place to control vehicle movement on their farms, including having a car parking location away from animal areas and having physical barriers to the animal areas. They also reported that control farms were more likely to allow dogs to accompany staff into livestock areas than case farms.

In their case-control study of cattle farms in Pakistan Ali et al. did not identify an association between FMD risk and equipment sharing based on a univariable *p*-value. However, Seifu et al. reported that 4.2% of surveyed Ethiopian dairy farmers who were “aware of FMD” did not share equipment as a means of preventing FMD spread (23, 33).

#### 3.4.4 Disinfection

Eleven reports discussed disinfection measures for FMD at the farm level, including those targeting clothes and footwear, vehicles, barns, premises, and various other targets. Four reports mentioned disinfection strategies targeting clothing and footwear which included descriptions and the effectiveness of disinfecting worker clothing or utilizing boot dipping stations. Eight reports mentioned disinfection strategies targeting vehicles including descriptions of general vehicle disinfection, disinfection of wheels and tires specifically, and a description of utilizing vehicle disinfection equipment. Two reports described disinfectant strategies targeting barns specifically. One report described general site disinfection strategies, and three reports described disinfection strategies for other targets (e.g., staff, equipment, and supplies). The disinfectant product used was often not specified within the reports, but when reported, disinfectants included Virkon S (38), lime and ash water (35), slaked lime, formalin, and “crushed phenyl tabs” (23), organic acids, formaldehyde, and potassium permanganate (29). The number of reports where unique disinfection biosecurity measures were implemented by their reported effectiveness is summarized in Table 7. Expanded descriptions for disinfection biosecurity measures and their effectiveness are reported in Supplementary Table 4.

**Table 7:** Number of reports where unique disinfection biosecurity measures were implemented by their reported effectiveness.

Biosecurity measure	Number of unique reports				
	Beneficial	Detrimental	Neutral	Effect not determined	Total (*n* = 11)
Clothing	0	0	1	1	2
Foot dips	0	0	2	0	2
Disinfection of vehicles	1	0	1	1	3
Wheels and tires	0	0	2	2	4
Possession of vehicle disinfection equipment	0	0	0	1	1
Barn and animal housing	1	0	0	1	2
Regular disinfection (target not specified)	0	0	1	0	1
Disinfection dip at entrance (target not specified)	0	0	0	1	1
Disinfection of researcher equipment	0	0	0	1	1
Disinfection of staff	0	0	0	1	1
General disinfection of supplies (not specified)	0	0	0	1	1
Sanitizing efforts in nomadic herds	0	0	0	1	1

A single report may discuss variable outcomes for individual biosecurity measures. Therefore, reports may belong to more than one effect category per row.

Two reports discuss disinfecting clothing during FMD outbreaks. Kass, et al. simply reported that staff clothing was disinfected during the 1982 FMD outbreak in Soviet Estonia without giving further details regarding products used, methods, or effectiveness. Wee, et al. described spray disinfectant being applied to the jeans of a worker who participated in culling activities during the 2002 FMD outbreak in Korea. In the report the authors speculate that the disinfection process used was insufficient to prevent subsequent contamination of the worker's vehicle interior. Byomi and Ellis-Iverson et al. both report the use of boot dips, but neither provide evidence of the practice's effectiveness in reducing disease transmission (21, 35–37).

Eight reports described vehicle disinfection strategies (21, 29, 34, 35, 37–40). General vehicle disinfection was reportedly used as part of a successful site decontamination process on a sow farm in Asia (29). However, general vehicle decontamination efforts either had no association with FMD transmission risk (34) or were not evaluated (39) in other reports. Four reports mention the use of decontamination strategies specific to vehicle wheels and/or tires (e.g., wheel dips, sprays, or washes, or disinfection mats or baths), but these strategies either appeared not to be associated with decreasing FMD transmission risk (21, 37) or were not evaluated for effectiveness (35, 38). Lee et al. (40) reported the presence of vehicle disinfection equipment on 61% of swine farms and 35% of cattle farms infected during FMD outbreaks between 2014 and 2019 in South Korea, but no details were given as to their use or effectiveness. Similar to other disinfection reports, details regarding methods, protocols, or products used during specific vehicle disinfection efforts as well as estimates of effectiveness are lacking.

The disinfection of barns was described in two reports. Poulin and Christianson (29) described the cleaning of the entire barn space through pressure washing and organic acid rinses, emptying of the manure pit, changing lightbulbs, and fumigation of the entire barn with formaldehyde and potassium permanganate during the successful decontamination of a swine farm in Asia. Kass, et al. reported that barns were disinfected with lime and ash water during the 1952 FMD outbreak in Soviet Estonia, but no further detail was given about the method of application or estimated effectiveness (35).

In their case control study evaluating a 2019 FMD outbreak in Pakistan, Ali et al. (23) discussed two strategies for general premises disinfection. In a univariable model, they did not identify an association between disease risk and the farms that reported performing disinfection regularly, and while no consistent disinfection protocols or products were evaluated, the most commonly reported disinfectants included slaked lime, formalin, and “crushed phenyl tablets (phenylephrine hydrochloride)”. The authors also reported that a small proportion of the total enrolled farms (7/128, 5.5%) utilized disinfection dips at the farm entrance, but did not evaluate the measure for effectiveness, nor were any further details provided regarding disinfection target, protocol, or products used (23).

Three reports discussed other disinfection strategies or targets (e.g., staff, equipment, and supplies), but none were evaluated for effectiveness. Mielke, et al. mentioned the application of Virkon S to researcher's shoes and equipment during an environmental sampling study; however, the timing and intended purpose of the application within the study design was unclear. Kass, et al. reported regular disinfection of staff and supplies being implemented during the 1982 FMD outbreak in Soviet Estonia but did not detail disinfection methods or what supplies were targeted. DiPietro, et al. reported that a small proportion of surveyed nomadic herders from Mongolia indicated that they utilized “sanitizing efforts” to prevent or respond to FMD (3/104), but did not provide details about the reported efforts (27, 35, 38).

## 4 Discussion

Numerous biosecurity practices identified in this review are plausibly effective, and the data presented here do not undermine that intuitive sense. However, the available data are not sufficient to support or refute their effectiveness or the specific critical control points necessary for effective implementation. We propose that field biosecurity measures be assessed for potential usefulness utilizing three practical criteria. First, the proposed measure must plausibly interrupt a transmission route of the disease of interest. Second, the proposed measure should be effective in the field at mitigating disease risk. Third, the measure should ideally be suitable for practical and economic implementation in the field setting. The biosecurity measures identified in this review, along with other recommended best practices are generally accepted as meeting the first criterion, as evidenced by the recommendation of their implementation in guidance documents (41). These measures also may plausibly be thought to mitigate risk, and in some cases, may not be overly onerous or expensive. However, reported data that support the second and third criteria are largely lacking.

From this review, no biosecurity measures that appeared in more than one report were identified as being consistently effective at mitigating disease risk in a field setting. Showering and changing outerwear was demonstrated to be consistently effective for preventing FMD transmission in a laboratory setting; however, no reports were identified to support the effectiveness of this biosecurity measure in the field (19, 20). Handwashing and changing outerwear were also demonstrated to have variable efficacy in a laboratory setting, depending on viral strain and susceptible species, but again, no reports were identified to support this measure's effectiveness in the field (19, 20). Utilizing spatial separation as a management strategy for groups of livestock within herds (22, 29, 30) and vehicle disinfection (29, 34, 39) were reported to be inconsistently effective at mitigating disease risk in field settings. Managing feral animal farm access and cleaning barns through pressure washing, organic acid rinses, and fumigation with formaldehyde and potassium permanganate were described as being effective for mitigating disease risk in a single report (29), but no estimate of effectiveness was given in other reports where these measures were mentioned (31, 32, 35). Using sealed containers or waterproof sheets during carcass transport (34), having a physical barrier to farm access (37), and having designated visitor parking away from animal areas (37) were all reported to be effective for mitigating disease risk in one report each. While plausibly effective, more evidence is needed to achieve a consensus regarding the effectiveness of these biosecurity measures. Allowing dogs to accompany farm staff (37) was also reportedly effective for mitigating disease risk but may exemplify a spurious relationship. None of the biosecurity measures describing the utilization of PPE or individual management were reported as being effective for mitigating disease risk (22, 31–33, 35–37). Furthermore, the reporting detail regarding PPE use in most of the included reports was not sufficient for making meaningful comparisons of PPE effectiveness among reports. Additionally, in the Wee et al. report from the 2002 outbreak in Korea, the PPE failure was arguably a failure of compliance since clothes worn under the PPE were worn home without sufficient disinfection (36). Notably, the practice of isolating clinically ill animals was reported to be ineffective for mitigating disease (23) and also implicated in exacerbating disease spread (21, 22). The same issues of insufficient reporting of protocols and poor implementation of plausible biosecurity measures that were identified regarding reports on PPE effectiveness, were also likely present in animal isolation reports. The inconsistent and limited evidence to support the effectiveness of specific biosecurity measures in field settings identified through this review hampers the ability of animal health officials and animal caretakers to plan for and respond appropriately during an FMD outbreak.

Because of its highly contagious nature and trade impacts, researchers are limited in their ability to study FMD in field-trial settings. In countries free of the disease such as the U.S. and United Kingdom (U.K.), high-containment bio-laboratories are needed to investigate FMD. However, these controlled environments cannot completely replicate all variables present in field settings. We identified two laboratory studies that experimentally evaluated the efficacy of biosecurity measures for preventing the transmission of two different FMD strains (19, 20). In both studies, researchers demonstrated that implementing certain biosecurity measures for animal caretakers can reduce the risk of indirect FMD transmission. While showering and changing outerwear appeared to be the most effective biosecurity measure evaluated, hand washing and changing outerwear was more effective than not implementing any biosecurity measures. The efficacy of hand washing and changing clothes in preventing the transmission of FMD varied based on FMD strain evaluated, susceptible species, and outcome used to assess FMD infection status. While useful in understanding transmission and biosecurity in high containment, the limitations of the laboratory environment make estimating the actual impact of handwashing, changing outwear, or complete showers in the field difficult. These laboratory results should be extrapolated to the field with caution as the incomplete implementation of biosecurity in the field setting could create a false sense of security in disease prevention. In a field setting, protocols often do not maintain the same level of stringency observed under laboratory conditions and can undergo protocol drift which can impact the protocol's actual effectiveness. An example of such protocol drift is often seen in milk producers in the U.S. with daily tasks such as preparing teats for milking (42). Additionally, while cattle were the most commonly reported species in the field biosecurity measures reviewed here, no cattle were included in the laboratory studies identified through this review. Furthermore, only two strains of FMD were investigated in the laboratory studies. While this is a strength for assessing the impact of interventions for those strains, it limits the application of the study findings to field settings. Infectivity varies among FMD strains which could impact biosecurity measure effectiveness (43). Consensus is also needed regarding which animal disease outcomes should be assessed to determine whether biosecurity measures are effective. The choice of measured outcome (e.g., clinical signs, viremia, seroconversion) should be relevant to claims made regarding biosecurity efficacy/effectiveness (e.g., prevention of disease, transmission, infection, exposure).

In addition to the previously mentioned lack of consensus regarding measured outcomes and efficacy/effectiveness criteria, several other reporting issues precluded further analysis of the effectiveness of farm-level biosecurity measures against FMD. Few reports were identified that quantified the effectiveness of specific biosecurity measures. Most reports included in this review were observational studies or descriptive reports with small sample sizes and simple descriptions of biosecurity measures implemented without an assessment or estimate of effectiveness. Additionally, the implemented biosecurity measures, whether assessed or not, were generally not described in sufficient detail to allow a clear understanding of the practice implemented. These limitations increase the potential for the reported results to be type 1 and type 2 errors. A type 1 error may have occurred in reports where the authors are crediting the biosecurity measure with effects that were actually a result of chance or good luck, such as in the report that found dogs accompanying staff to work to be protective (37). Additionally, type 2 errors may be present in reports that did not find a biosecurity measure to be effective when other studies, and biological plausibility suggest otherwise. For example, many reports referred to isolation or quarantine of animals but very few defined the distance the animals were quarantined from others or the duration of the quarantine. Isolation was not reliably associated with decreased risk, in some cases it was associated with increased risk; however, the details of how isolation was implemented are not sufficient to sort out what practices were actually reported or evaluated. Unclear and insufficient reporting is also a limitation for evaluating disinfection biosecurity measures. Future reports should include adequately detailed descriptions of implemented biosecurity measures or protocols to allow for subsequent comparisons. To accomplish adequate reporting, standardized reporting guidelines to evaluate biosecurity measure effectiveness would be helpful. The STROBE-Vet Statement (Strengthening the Reporting of Observational Studies in Epidemiology—Veterinary Extension) provides reporting guidance for observational studies conducted in all animal species (44). Additionally, outbreak investigation reporting guidance has been developed for use during human disease outbreaks that could potentially be modified for veterinary use (45, 46). In addition to reporting guidance to standardize study design reporting uniformity and transparency, consensus regarding reporting guidance for defining biosecurity measures, describing biosecurity measure protocol implementation, and assessing biosecurity measure outcome effectiveness will also be necessary to allow the comparison of biosecurity measure effectiveness among reports.

Field based research on biosecurity is a fundamentally difficult endeavor. Unlike experimental research in a controlled laboratory setting, exposure risk and magnitude is unknown so the study population of farms are a mix of those exposed and infected (biosecurity failure), exposed and uninfected (biosecurity success), and those unexposed (biosecurity not tested). For FMD, rare exposure between outbreaks provides few opportunities to test biosecurity practices, while assessment during outbreaks is difficult due to the demands of outbreak management and the variable farm risk. Individual farm risk may differ dramatically from farms with high risk which may overwhelm even good biosecurity to farms with low risk which may avoid infection in spite of poor biosecurity.

Robust assessment of field-biosecurity may be best served by long term prospective cohort studies of farms with well-defined and implemented biosecurity measures followed over time and through outbreaks to assess effectiveness. The necessary number of farms enrolled would be large to account for the inherent variability in farm exposure. However, the level of biosecurity required to be effective against FMD is likely not practical or economic for everyday biosecurity. As such, establishment of well-defined enhanced biosecurity plans for “just in time” implementation at the start of a local outbreak may be an achievable alternative. This would require development of a cohort of motivated and willing producers for education on effective implementation of biosecurity measures. Immediately at the onset of an outbreak, collection of data on defined biosecurity implementation (daily records of implementation and deviations), local incidence risk, and active surveillance for disease occurrence would be required. Once disease was identified at a farm, active surveillance within the farm would be necessary to assess any biosecurity measures meant to control disease spread on the farm. These would be resource intensive studies for field evaluation of effective biosecurity measures. Application of target trial emulation practices would be necessary to strengthen the study design and subsequent results (47). Future research in the form of case-control studies may also be helpful but will be hampered if well-defined and pre-planned biosecurity practices are not evaluated and substantial effort devoted to assuring control farm status.

It is also critical that the results of the biosecurity trials and research are adopted by the producers. Recent research has shown that currently, adoption of biosecurity planning by U.S. producers is low (48). In their 2021 survey, Pudenz, et al. found that only 4% of U.S. cow-calf producers and 7% of feedlots have a biosecurity plan. This is not surprising given the rarity of large-scale national disease outbreaks affecting U.S. cattle in recent years. Producers cannot be expected to implement biosecurity measures that have no positive economic return. In FMD free countries the path toward better biosecurity should prioritize biosecurity measures that are effective against endemic diseases to demonstrate the value of utilizing biosecurity to decrease disease risk. Despite the low level of biosecurity adoption in the U.S. cattle industry, FMD preparation plans continue to be developed in various academic, state, and federal settings.

Reports included in this scoping review were limited to those published in English which may have eliminated some relevant reports from FMD-endemic areas. The elimination of a search update from the CAB database, may also have eliminated a few relevant reports; however, since no reports included from the original search were unique to the CAB database, its elimination from the search update is unlikely to be a major source of bias. Additionally, while only reports from peer-reviewed sources were considered for inclusion in this review, which served to decrease search time, conducting the topic search as part of a larger project ultimately increased the time necessary to complete the screening process. These methodology choices impacted the timeliness of review completion and could be decreased with a more limited search strategy. Lastly, the lack of defined criteria for what is or is not considered a farm-level biosecurity measure and what does or does not constitute effectiveness also presented challenges for data charting that impacted the timeliness of the review. Having clearly defined criteria for these and other terms would enhance repeatability of reviews focusing on the effectiveness of biosecurity measures.

## 5 Conclusion

The reports included in this review provide insight regarding biosecurity measures used to manage FMD in a large variety of geographic locations and farm types. The reports represent measures that producers are using in the field to mitigate the introduction and spread of FMD at the farm level globally. While the challenges of evaluating biosecurity measure effectiveness identified in this review hamper U.S. preparedness efforts, the lack of consensus and data to support the effective implementation of biosecurity measures also impacts farms globally.

A number of lessons learned are apparent from this review. There were various weakly evidenced descriptions of the efficacy or effectiveness of interventions that give some credibility to support the use of the reported biosecurity measures. However, it remains difficult to extrapolate the effectiveness of the reported measures to other regions and production types given the differences between FMD-endemic and FMD-free regions in disease spread and animal production systems. The scope of this review was focused on measures that could influence U.S. decision making but the information gap remains globally. Additionally, incomplete reporting regarding the description and implementation of many biosecurity measures contributes to the imprecision of biosecurity measure effectiveness estimates. Future reports of biosecurity measures implemented during FMD outbreaks would benefit from the utilization of current reporting guidance as well as the development of standardized reporting guidance for biosecurity descriptions, biosecurity measure implementation protocols, biosecurity effectiveness outcome assessment, and animal disease outbreak investigations. Accurate descriptions of biosecurity measure implementation and protocols are critical to allow the comparison of effectiveness among reports.

While current FMD preparation and response plans promote biosecurity measures that are “best practices” and may decrease the likelihood of FMD introduction or spread, clear evidence to support the effectiveness, or lack of effectiveness, of the recommended biosecurity measures is currently lacking in peer-reviewed literature. Ultimately, more studies, both in laboratory and field settings, in the U.S. and beyond, are necessary to validate field use of biosecurity measures with demonstrated laboratory efficacy, to build consensus regarding the effectiveness of biosecurity measures currently in use, and to inform which biosecurity measures have effectiveness that may vary depending on the outbreak strain of FMD and species affected. Better reporting of implemented or evaluated biosecurity measures in the literature will allow producers, animal health officials, and veterinarians to make informed decisions in the face of an outbreak.

## Data Availability

The original contributions presented in the study are included in the article/Supplementary material, further inquiries can be directed to the corresponding author.
