# Supplementary material for: A scoping review of farm-level biosecurity measure effectiveness against foot-and-mouth disease to inform planning and preparedness efforts in the United States
Source: Front Vet Sci. 2026 Jun 8;13:1819419. doi: 10.3389/fvets.2026.1819419 (PMC13284146; doi:10.3389/fvets.2026.1819419)
Supplement: Supplementary file 1 [file Table_1.docx]

**Supplemental Table 1: Livestock and product management: description and effect of biosecurity measures for managing livestock, and livestock products at the report level**

| **Biosecurity measure** | **Description of implementation and effectiveness** | **Reference** |
| --- | --- | --- |
| **Isolation, Quarantine, Animal Separation (n=13)** | | |
| Isolation of infected animals from the rest of the herd | **Detrimental** |  |
|  | Authors report the presence of “isolation pens located near highway and not separated from other farm units… contributed to the spread of the disease”. | Byomi, 2015 |
|  | Authors reported that groups of cattle were moved to an isolation paddock after identification of the index case in each group. Authors suggest that the movement of infected cattle led to exposure and subsequent infection of other cattle on the farm, contributing to outbreak propagation. | Lyons, et. al, 2015 |
|  | **Neutral** |  |
|  | 53/64 case farms and 44/64 control farms reported isolating sick animals. This practice was significant in a univariable model (p = 0.091), but not retained in the final multivariable model. | Ali, et. al, 2022 |
|  | **Not Evaluated** |  |
|  | Farmers reported “separating sick animals from unaffected animals upon noticing clinical signs consistent with FMD”.  No isolation protocol was specified.  Authors noted that “disinfection of clothing and equipment after treating FMD-infected animals was rarely practiced”. | Nampanya, et. al, 2012 |
|  | 31.7% (20/63) households surveyed reported isolating clinically unwell animals until recovered. Biosecurity measure was not assessed for effectiveness due to significant collinearity with other variables. | Miller, et. al, 2018 |
|  | 32% (9/28) of cattle owners who reported having at least one case of FMD in the previous six months reported using isolation as a disease management strategy. | Shurbe, et. al, 2022 |
|  | Nomadic herders reported that isolation of infected animals is implemented during outbreaks. | DiPietro, et. al, 2023 |
|  | Smallholder cattle farmers (n=240) reported always (52.1%), sometimes (25.4%), or never (22.5%) separating sick from healthy cattle.  No isolation protocol was specified. | Young, et. al, 2016 |
| Separation of animal groups on farm | **Beneficial** |  |
|  | Pens of immune animals were an “effective barrier” against within-herd FMD transmission. | Hutber and Kitching, 2000 |
|  | Apparently healthy piglets were transferred to a new farm and never became clinical or tested positive for FMD. | Poulin and Christianson, 2006 |
|  | Keeping small ruminants on a separate grazing pasture “a few kilometers” from the cattle sufficient to prevent contact between them. No clinical FMD cases were reported in the small ruminants. | Lyons, et. al, 2015 |
|  | **Neutral** |  |
|  | Physical and spatial boundaries sufficient to slow but not prevent within-herd FMD transmission. | Hutber and Kitching, 2000 |
|  | Separation of clinically healthy sows from infected farm not sufficient to prevent subsequent outbreaks of clinical disease. | Poulin and Christianson, 2006 |
| Isolation, quarantine, or inspection of incoming stock | **Neutral** |  |
|  | No significant difference in disease risk found between the 2/64 case farms and 3/64 control farms that reported utilizing a quarantine measure for new herd introductions (univariable p-value = 0.763). Quarantine protocol not defined. | Ali, et. al, 2022 |
|  | **Not Evaluated** |  |
|  | 72.0-88.7% of survey respondents inspected and/or isolated incoming stock.  No specific protocols were defined, actions included inspection for disease, isolation, and certification of inspection by veterinary professional. | Manyweathers, et. al, 2020 |
|  | 64.9-84.1% of survey respondents inspected and/or isolated incoming stock.  No specific protocols were defined, actions included inspection for disease, isolation, and certification of inspection by veterinary professional. | Manyweathers, et. al, 2021 |
|  | Smallholder cattle farmers (n=240) reported always (26.3%), sometimes (25.8%), or never (47.9%) isolating incoming stock for 2 weeks. | Young, et. al, 2016 |
| Closed herd | **Not Evaluated** |  |
|  | 3.1% of 93 dairy farmers who were “aware of FMD” reported that they practiced not bringing new cattle onto their farms as a prevention measure.  6.3% of 93 dairy farmers who were “aware of FMD” reported that they prevented other cattle from entering their farms.  9.6% of 93 dairy farmers who were “aware of FMD” reported that they practiced keeping cattle within the farm compound. | Seifu, et. al, 2023 |
|  | 19.2% of 240 smallholder cattle farmers reported keeping their cattle from having direct contact with other farmers’ cattle as a prevention measure.  78.3% of 240 smallholder cattle farmers reported keeping their cattle in a fenced area as a prevention measure. | Young, et. al, 2016 |
| **Animal Products and Wastes (n=2)** | | |
|  | **Beneficial** |  |
| Carcass transport | Covering carcasses with waterproof sheets or using disinfected trucks with sealed containers to transport carcasses from farms to burial sites was not significantly associated with increased transmission risk in a multivariable model (Operational burial sites within 500m p-value = 0.98; Operational transportation roads within 200m p-value = 0.34). | Hayama, et. al, 2015 |
|  | **Not Evaluated** |  |
| Carcass disposal | Dead calves were burned on bonfires. | Kass, et. al, 2022 |
| Feces | Manure from sick cows was stored and not immediately used for fertilizer. |  |
| Milk | Milk from infected cattle was added to manure for disposal or pasteurized and fed to other animals. |  |

**Supplemental Table 2: Personal protective equipment and individual management: description and effect of biosecurity measures for managing farm personnel and visitors at the report level**

| **Biosecurity measure** | **Description of implementation and effectiveness** | **Reference** |
| --- | --- | --- |
| **Personal Protective Equipment (n=3)** | | |
| Clothing | **Neutral** |  |
|  | Use of disposable work suit (material not specified) during culling activities likely insufficient to prevent viral contamination of clothing. | Wee, et. al, 2008 |
|  | No association identified between providing farm staff with farm-specific clothing (material not specified) and being a case farm (univariable p-value = 0.47). | Ellis-Iverson, et. al, 2011 |
|  | **Not Evaluated** |  |
|  | Use of protective clothing (material not specified) by stockpersons and other employees on a dairy farm was reported. Farm was located in a district that did not have an FMD case during the 1982 outbreak. | Kass, et. al, 2022 |
| Footwear | **Neutral** |  |
|  | No association identified between providing farm staff with farm-specific footwear (material not specified) and being a case farm (univariable p-value = 1.00). | Ellis-Iverson, et. al, 2011 |
|  | **Not Evaluated** |  |
|  | Use of boot covers during culling activities was reported. Boot covers and boots were destroyed following activities, and worker returned home without footwear. | Wee, et. al, 2008 |
| Gloves | **Not Evaluated** |  |
|  | Workers reportedly wore rubber gloves while milking cows with FMD lesions. | Kass, et. al, 2022 |
| **Individual Management (n=6)** | | |
| Restricting visitor access to farms | **Not Evaluated** |  |
|  | 25.4% of surveyed beef producers always restrict visitor farm access.  23.1% of surveyed beef producers never restrict visitor farm access and 39.3% of respondents never require visitors to undergo specific biosecurity practices. | Manyweathers, et. al, 2020 |
|  | 32.3% of surveyed sheep producers regularly restrict visitor farm access.  53.6% of surveyed sheep producers did not require visitors to follow biosecurity practices. | Manyweathers, et. al, 2021 |
|  | 2.1% of 93 dairy farmers who were “aware of FMD” reported that they practiced not allowing visitors on their farms. | Seifu, et. al, 2023 |
| Farm lockdowns | **Not Evaluated** |  |
|  | During both the 1952 and 1982 FMD outbreaks farm personnel were confined to the infected premises for extended periods. Farm access by non-employees was prohibited. Movement restrictions were enforced by militia and breaches were punishable by imprisonment. | Kass, et. al, 2022 |
| Bathing | **Not Evaluated** |  |
|  | Use of public bath reported as part of decontamination protocol. No specific protocol reported. | Wee, et. al, 2008 |
| Minimizing cross-contact | **Not Evaluated** |  |
|  | Stockmen assigned to single cattle groups during the outbreak period. | Lyons, et. al, 2015 |

**Supplemental Table 3: Fomite management: description and effect of biosecurity measures for managing fomites at the report level**

| **Biosecurity measure** | **Description of implementation and effectiveness** | **Reference** |
| --- | --- | --- |
| **Non-livestock (n=4)** | | |
| Feral animal management | **Beneficial** |  |
|  | Rodent control program strengthened to prevent mechanical reintroduction as one component of a successful FMD eradication and site decontamination process. | Poulin and Christianson, 2006 |
|  | **Not Evaluated** |  |
|  | 18.7% of surveyed beef producers never, rarely, or occasionally control feral animals on their farm (vs most of the time or always) | Manyweathers, et. al, 2020 |
|  | 29.6% of surveyed sheep producers have control plans for feral animals on their farm. | Manyweathers, et. al, 2021 |
| Dog Permission | **Beneficial*** |  |
|  | Allowing dogs to accompany staff onto livestock areas was less likely on case farms than control farms (univariable p-value = 0.05).  None of the 5 case farms allowed dogs, 12 of 22 control farms did. | Ellis-Iverson, et. al, 2011 |
| **Vehicles (n=2)** | | |
| Designated parking | **Beneficial*** |  |
|  | Case farms were less likely than control farms to have visitor car parking areas away from animal areas (univariable p-value = 0.03). | Ellis-Iverson, et. al, 2011 |
| Barrier to vehicle entry | **Beneficial*** |  |
|  | Case farms were less likely than control farms to have gates/fences at the entrance to livestock areas (univariable p-value = 0.14). | Ellis-Iverson, et. al, 2011 |
| **Equipment (n=2)** | | |
| Equipment management | **Neutral** |  |
|  | No significant association with disease risk found between the 1/64 case farms and 2/64 control farms that reported sharing equipment (univariable p-value = 0.56). | Ali, et. al, 2022 |
|  | **Not Evaluated** |  |
|  | 4.2% of 93 dairy farmers who were “aware of FMD” reported that they practiced not sharing equipment as an FMD prevention measure. | Seifu, et. al, 2023 |

*Association of risk factor and case status were reported by authors to be significant based on a univariable p-value

**Supplemental Table 4: Disinfection: description of on-farm disinfection measures and their effectiveness to control FMD introduction or spread at the report level**

| **Disinfection target or strategy** | **Description of implementation and effectiveness** | **Reference** |
| --- | --- | --- |
| **Clothing and Footwear (n=4)** | | |
| Clothing | **Neutral** |  |
|  | Jeans worn under a disposable work-suit during culling event sprayed with “disinfectant” and wrapped in vinyl during personnel decontamination process. Process appears to be insufficient for preventing contamination of the interior of the worker’s vehicle. | Wee, et. al, 2008 |
|  | **Not Evaluated** |  |
|  | During the 1982 FMD outbreak, the staff’s clothing was disinfected on a regular basis (product or protocols not specified). | Kass, et. al, 2022 |
| Foot Dips | **Neutral** |  |
|  | Use of foot dips not sufficient to prevent FMD outbreak on farm (product utilized not specified). | Byomi, 2015 |
|  | No association identified between utilizing boot dips (product and protocol not specified) and being a case farm (univariable p-value = 0.30). | Ellis-Iverson, et. al, 2011 |
| **Vehicles (n=8)** | | |
| Vehicles | **Beneficial** |  |
|  | The internal feed truck was washed weekly (products and protocol not specified) during a successful site decontamination process. | Poulin and Christianson, 2006 |
|  | **Neutral** |  |
|  | Disinfection of vehicles transporting carcasses from farms to burial sites (products and protocol not specified) was reported in conjunction with other biosecurity measures. The presence of transportation roads within 200 meters or burial sites within 500 meters was not significantly associated with FMD transmission risk to farms (Operational burial sites within 500m p-value = 0.98; Operational transportation roads within 200m p-value = 0.34). | Hayama, et. al, 2015 |
|  | **Not Evaluated** |  |
|  | 86.1% (385/447) of both case (n=140) and control (n=307) farms reported using disinfectant for vehicles (product not specified). | Sansamur, et. al, 2020 |
| Wheels and tires | **Neutral** |  |
|  | Use of wheel dips not sufficient to prevent FMD outbreak on farm (product utilized not specified). | Byomi, 2015 |
|  | No association identified between utilizing wheel washes (product and protocol not specified) and being a case farm (univariable p-value = 1.00). | Ellis-Iverson, et. al, 2011 |
|  | **Not Evaluated** |  |
|  | During both the 1952 and 1982 FMD outbreaks, disinfection mats were reportedly used on the roads and at farm entrances for vehicles to drive over. Drivers would also exit and disinfect shoes.  Products used were not specified. | Kass, et. al, 2022 |
|  | During the 1982 FMD outbreak, a disinfection bath was utilized on a dairy farm that would completely wet the wheels and tires of feed delivery vehicles. The farm was located in a district that did not have an FMD case during the outbreak. |  |
|  | Authors report that a solution of one tablet of Virkon S diluted in 500 mL of water was sprayed on car tires during an environmental sampling study. | Mielke, et. al, 2023 |
| Vehicle disinfection equipment | **Not Evaluated** |  |
|  | 61.2% (126/206) of swine farms infected between 2014 and 2019 reported having vehicle disinfection equipment.  35.3% (6/17) of cattle farms infected between 2014 and 2019 reported having vehicle disinfection equipment. | Lee, et. al, 2021 |
| **Barns (n=2)** | | |
| Barns | **Beneficial** |  |
|  | During a successful site decontamination, swine barns and manure pits were thoroughly cleaned and rinsed with organic acids. After which, they were then fumigated with formaldehyde and potassium permanganate. | Poulin and Christianson, 2006 |
|  | **Not Evaluated** |  |
|  | During the 1952 outbreak, barns were reportedly disinfected with lime and ash water. | Kass, et. al, 2022 |
| **General site (n=1)** | | |
| Regular disinfection | **Neutral** |  |
|  | No significant association with disease risk found between the 12/64 case farms and 15/64 control farms that reported performing disinfection regularly (univariable p-value = 0.564).  The most usual materials used for disinfectants were reported to be slaked lime, formalin, and crushed phenyl tablets. | Ali, et. al, 2022 |
| Disinfection dip at entrance | **Not Evaluated** |  |
|  | 7/128 (5.5%) of the total case and control farms had a disinfection dip at the farm entrance. Specific products and dip use were not specified. | Ali, et. al, 2022 |
| **Other (n=3)** | | |
| Researcher equipment | **Not Evaluated** |  |
|  | Authors report that a solution of one tablet of Virkon S diluted in 500 mL of water was sprayed on researcher equipment during an environmental sampling study. | Mielke, et. al, 2023 |
| Staff | **Not Evaluated** |  |
|  | During the 1982 FMD outbreak, farm staff were reportedly disinfected on regular basis (product or protocols not specified). | Kass, et. al, 2022 |
| Supplies | **Not Evaluated** |  |
|  | During the 1982 FMD outbreak, farm supplies were reportedly disinfected on regular basis (product or protocols not specified). | Kass, et. al, 2022 |
| Sanitizing Efforts | **Not Evaluated** |  |
|  | “Sanitizing efforts” were reportedly used by 3/104 surveyed nomadic herders to prevent or respond to FMD. | DiPietro, et. al, 2023 |
